# Meeting OCD face-to-face: Preliminary findings from an avatar-based dialogue intervention

**DOI:** 10.1177/20552076261434822

**Published:** 2026-04-02

**Authors:** Lara Wille, Luzie Lohse, Steffen Moritz, Josephine Schultz, Swantje Borsutzky, Amir H. Yassari, Lise Mariegaard, Ditte Lammers Vernal, Sanne Helene Bekker, Louise Birkedal Glenthøj, Franziska Miegel, Lena Jelinek

**Affiliations:** 1 Department of Psychiatry and Psychotherapy, 163303 University Medical Center Hamburg-Eppendorf, Hamburg, Germany; 2 86985 VIRTU Research Group, Mental Health Center Copenhagen, Copenhagen University Hospital – Mental Health Services CPH, Copenhagen, Denmark; 3 Department of Psychology, University of Copenhagen, Copenhagen, Denmark; 4 Psychiatry, 193588 Aalborg University Hospital, North Denmark Region, Aalborg, Denmark; 5 OPUS, 532071 Psychiatry in Region North Jutland, Aalborg, Denmark

**Keywords:** OCD, VRT, insight, externalization, avatar

## Abstract

**Background:**

Obsessive-compulsive disorder (OCD) is a chronic, debilitating condition typically treated with cognitive-behavioural therapy (CBT) and exposure and response prevention (ERP). While effective, these approaches are often met with hesitation, as many patients struggle to engage in exposure-based treatment, and relapse remains a significant challenge. Virtual Reality-assisted Therapy (VRT), initially developed for auditory hallucinations, offers a novel, immersive approach that may enhance insight by helping patients externalize their OCD symptoms. This perspective shift may increase engagement and motivation for ERP. This study evaluates the feasibility, safety, acceptability and preliminary treatment effects of VRT adapted for OCD (VRT-OCD), in which patients interact with a virtual avatar embodying their disorder.

**Methods:**

Eight inpatients with OCD received three weekly VRT-OCD sessions alongside standard care. Participants created personalized avatars representing their OCD and engaged in therapist-guided dialogues aimed at confronting their OCD. Assessments focused on feasibility, safety, acceptability, and preliminary treatment effectiveness (e.g. OC symptoms, insight, self-esteem).

**Findings:**

VRT-OCD did not show negative side effects and was well accepted by both patients and therapists. Participants reported an increased motivation for therapy and ERP.

**Interpretation:**

VRT-OCD appears to be a feasible, safe and well-accepted adjunctive treatment for OCD. Larger-scale studies are needed to validate these findings and further explore their potential to increase motivation for ERP.

**Funding:**

This research was not supported by third-party funding.

## Introduction

Obsessive-compulsive disorder (OCD) is a mental health condition – characterized by intrusive thoughts (obsessions) and repetitive behaviours (compulsions) – that affects millions worldwide, severely impacting quality of life and functioning.^1,2^ Exposure and response prevention (ERP), a form of cognitive-behavioural therapy (CBT), is widely regarded as the gold standard for OCD treatment and recommended as primary treatment.^3^ While both CBT and ERP are effective,^4^ around 25% of patients discontinue treatment prematurely,^5^ and relapse rates range between 17% and 59%.^6,7^ These challenges highlight the critical need for additional treatment options. One contributing factor to drop-out or relapse may be that some patients experience their OCD as an intrinsic part of their identity, hindering them from acknowledging their obsessions and compulsions as problematic.^8^ This perspective may reduce motivation to undergo treatment in the first place, especially regarding demanding treatment such as ERP.8 Virtual reality-assisted therapy (VRT), as an innovative add-on, could provide an engaging way to address this issue.

Originally developed for people with auditory hallucinations in psychosis,^9^ VRT enables users to create and interact with a virtual representation of their disorder, fostering a sense of agency and reducing its perceived control over them. Studies have shown VRT to be effective in improving people's coping with their auditory hallucinations (standing up to it) and even in reducing the frequency of auditory hallucinations.^10,11^ Just as people experiencing auditory hallucinations, people with OCD also feel controlled by their disorder – their OCD drives their behaviour, causing them to act according to their fears. An adapted version of VRT for OCD (VRT-OCD) would allow patients to challenge their OCD and regain a sense of control. Although obsessions in OCD do not constitute hallucinations per se, they frequently possess perceptual qualities. For example, many individuals with OCD report experiencing their obsessions in auditory, visual, or other sensory forms.^12^ The presence of such perceptual features suggests conceptual overlap with experiences previously targeted by VRT, supporting the rationale for adapting this intervention for OCD.

### How does VRT-OCD work?

**Figure 1.**
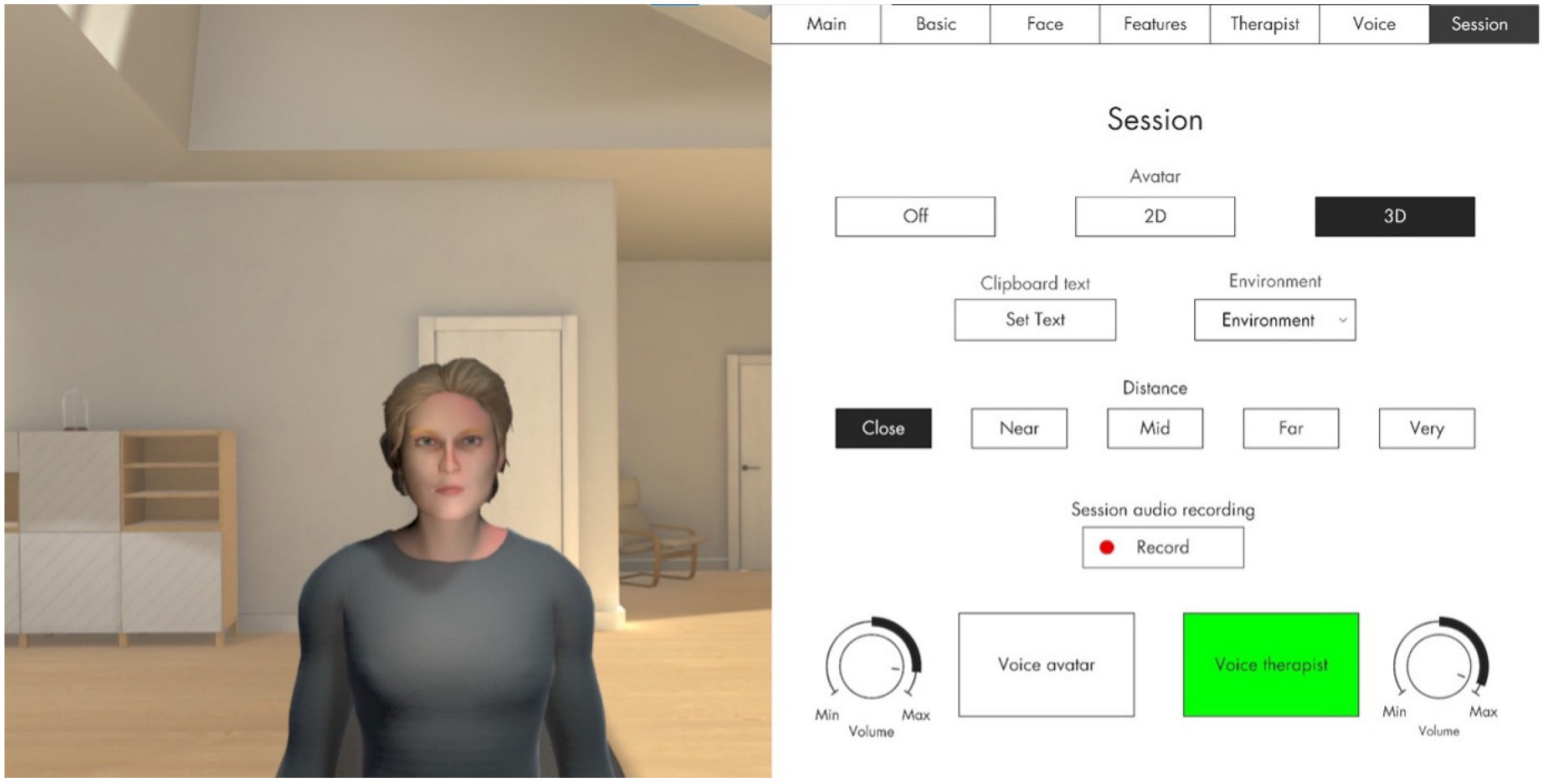
VR avatar.

*Note*: The therapist's view when using the software (developed by HekaVR). Left: the avatar from the patient's perspective. Right: the menu allowing them to switch between the avatar voice and their own.

In VRT-OCD, patients create a VR avatar that represents their OCD – this includes deciding on facial features and the avatar's voice (pitch, gender). They can enter a direct dialogue with their OCD using VR goggles and a (noise cancelling) headset. With a laptop and the respective software, the therapist can see the patient's view in VR as well as a dashboard (see Figure 1). The therapist uses a microphone and voice transformer to speak as the avatar, but also to switch back to their own voice to engage in the dialogue as the therapist (e.g. to motivate or to give instructions).

In session 1 (90 minutes), the therapist discusses the patient's most prevalent obsessions, using previously administered diagnostic tools (e.g. Yale-Brown Obsessive-Compulsive Scale [Y-BOCS]). Furthermore, therapist and patient discuss in which way the OCD is limiting the patient's daily life and to what extent they live according to their values. Psychoeducation on the therapy rationale is provided, and the patient creates the avatar representing their OCD, including voice modulation. Homework includes documenting statements of their obsessions verbatim and a worksheet on life values.

Session 2 introduces the first VR dialogue with the avatar. After reviewing the first session, the therapist and patient formulate verbatim statements the avatar will say in the dialogue and develop strategies for responding assertively while accepting uncertainty and engaging in valued action. The patient practices standing up to the avatar in a brief dialogue (5 minutes). The session concludes with a reflection and possibly strategy refinement and repetition of the dialogue (until the patient was able to stand up to the avatar). As homework, the patient gathers positive self-statements from a trusted person to use them in the following session.

Session 3 centres on increasing self-esteem. Positive self-statements are incorporated into a dialogue (10–15 minutes) in which the patient defends their values and identity against the avatar's challenges. As the patient demonstrates increased assertiveness, the avatar becomes less and less coercive. The session ends with a reflection on the therapeutic gains and the creation of action cards to support future coping.

The dialogue facilitates patients’ externalization of their OCD by encouraging them to view the disorder as a separate entity. The goal of VRT-OCD is a shift in perspective (‘you are not your OCD’), which is expected to increase psychological distance from obsessions and compulsions. This distancing may make it easier to appraise OCD-related cognitions and dysfunctional and to respond with greater agency.

Externalizing of distressing internal experiences or ‘parts’ of the self has been employed in established therapeutic approaches such as Gestalt therapy or Internal Family Systems.^13,14^ However, in OCD, empirical work directly assessing externalization processes, in particular in relation to insight, remains limited. Thus, the present adaptation of VRT for OCD was primarily guided by clinical considerations. We hypothesize that externalization and increased psychological distance may contribute to improvements in insight – the ability to see that one's obsessions and compulsions are unfounded or irrational.^15^ The process may also positively affect self-esteem,^16^ fear of self,^17^ and cognitive fusion^18^ – constructs theoretically linked to OCD symptomology.

Self-esteem is directly targeted in session 3 of VRT-OCD – something that has been adopted directly from the original VRT for people with auditory hallucinations.^9^ Attributing certain negative beliefs to the OCD (e.g. ‘I am only worthy and lovable when I make sure that I do not contaminate my loved ones’) and challenging these beliefs in dialogue with the avatar may help patients to update negative self-beliefs, potentially leading to an increase in self-esteem.

Fear of self refers to a patient's belief that they are a dangerous, immoral, or bad person, reinforcing the conviction that they need to control themselves to not harm others.^17^ By conceptualizing OCD as separate from the self and emphasizing the patient's values and positive personal characteristics, this belief can be challenged, supporting the development of a more compassionate and accurate self-concept.

Cognitive fusion is a state in which a person perceives their thoughts as the absolute truth and is not able to separate themselves from the thought.^18^ It seems likely that seeing one's OCD as a separate entity and learning to stand up to it (the OCD) may relate to changes in levels of cognitive fusion.

### Objective

Although the first results of VRT for people with auditory hallucinations are promising,^10^ we need to determine whether a transfer to OCD is feasible and safe. We conducted a small case study with eight participants (inpatients), evaluating feasibility, safety, acceptability, and preliminary treatment effects (e.g. OC symptoms, insight and self-esteem). Further, due to the small sample size, the results of this study must be considered with caution and may merely be used to inform future research, not to draw any final conclusions. In the long run, VRT-OCD may offer a way to increase patient motivation for ERP.

## Method

### Design

Participants were recruited from the anxiety and OCD ward of the University Medical Center Hamburg-Eppendorf (Germany). All patients received standard inpatient care, according to national guidelines, including ERP, medication, and group therapies (e.g. Metacognitive Training for OCD and occupational therapy) – avatar therapy was provided as an additional treatment.

All participants were pre-diagnosed with OCD by a clinician at intake according to the Mini International Neuropsychiatric Interview (MINI, version 7.0.2).^19^ At baseline of this study, a trained interviewer (bachelor's student) conducted diagnostic interviews to confirm OCD diagnosis as well as to determine OC symptom severity (Yale-Brown Obsessive Compulsive Scale, Y-BOCS) and degree of insight (Brown Assessment of Beliefs Scale**,** BABS). Further, participants filled out pen-and-paper questionnaires. Throughout the following three weeks, participants received weekly treatment sessions of VRT (60 minutes). At the beginning and end of each session, self-esteem and distance to the obsessions were rated by the participants. One week after the last session, participants were asked to fill out pen-and-paper questionnaires (assessing, e.g. feasibility, safety, acceptability and constructs related to OCD), and the Y-BOCS and BABS were re-assessed. Therapists were also asked to fill out pen-and-paper questionnaires after termination of the treatment, assessing the usability and suitability of VRT-OCD.

This study was approved by the local ethics committee of the Center for Psychosocial Medicine at the University Medical Center Hamburg-Eppendorf (Germany; *Lokale Psychologische Ethikkommission am Zentrum für Psychosoziale Medizin*, LPEK-0682).

### Participants

Eight participants were recruited following consultations with their respective therapists. Inclusion criteria were an OCD diagnosis according to the MINI,^19^ written informed consent, age between 18 and 75 years, sufficient command of the German language, and willingness to participate in VRT-OCD for three weeks, including the study assessments.

### Intervention

VRT-OCD is designed to help patients to reframe their experience of OCD and promote externalization of the disorder across three sessions. By using a visual avatar to represent the disorder, the approach seeks to empower patients to resist their obsessions and compulsions and enhance their insight into the nature of the condition. The intervention is guided by a manual which has been adapted from the manual for VRT for auditory hallucinations. The first session is used for psychoeducation and preparation (e.g. creating the avatar). The VR dialogues take place in sessions two and three. For the dialogues, the therapist speaks as the avatar using verbatim statements (obsessions) that the patient wrote down in advance. In session 2, patients practice standing up to their OCD, while the dialogue in session 3 focuses on self-esteem. All dialogues are prepared in-depth by the therapist and patient together. For a detailed description of the technical setup and sessions, please see Wille et al.^20^

In line with Acceptance and Commitment Therapy (ACT), VRT-OCD encourages living in accordance with personal values. Between sessions, patients complete homework assignments to clarify their values, fostering a new behavioural reference point, counteracting their obsessions.

## Instruments

To assess the feasibility of VRT-OCD, we used the Avatar Therapy Evaluation Scale for Therapists (ATEST), and the System Usability Scale (SUS).^21^ As another relevant feasibility criterion, we assessed the drop-out rate.^22^ Safety of the intervention was explored using the Inventory for the Balanced Assessment of Negative Effects of Psychotherapy (INEP).^23^ Acceptability and usability were measured using the Subjective Appraisal Rating Scale (SARS).^24^ OCD symptoms and related constructs were evaluated with the Yale-Brown Obsessive Compulsive Scale (Y-BOCS),^25^ the Brown Assessment of Beliefs Scale (BABS),^26^ the Rosenberg Self-Esteem Scale (RSE),^27^ the Fear of Self Questionnaire – Short Version (FSQ-SV),^28^ the Cognitive Fusion Questionnaire Deutsch (CFQ-D),^29^ and the Distance to Obsession Scale (DOS), which was developed specifically for this study (see supplement A). Additionally, in-session changes were assessed using the DOS in-session and a brief Self-Esteem in-session measure. Details on the instruments developed for this study can be found in the supplements (A).

### Statistical analyses

All measures assessing feasibility, safety, and acceptability (ATEST, SUS, INEP, SARS) are reported descriptively. All measures assessing preliminary treatment effects (Y-BOCS, BABS, RSE, FSQ, CFQ, DOS) are also reported descriptively, and effect sizes were calculated from pre- to post-treatment. In-session data (distance to obsessions and self-esteem), assessed immediately before and after each session, are also reported descriptively with accompanying effect sizes.

As assumptions of normality were not met for the variables measuring preliminary effectiveness, we analysed the symptom changes from baseline to post assessment by performing the Wilcoxon Signed-Rank Test to look at the effect sizes (Cohen's *d*)*.* The effect size was calculated using the z-scores with the following formula: $d=\frac{z}{\surd N}\;$ . Effect sizes were interpreted as d ≈ 0.2 small, d ≈ 0.5 medium, and d ≈ 0.8 large, following Cohen.^30^ Following the same approach, effect sizes were calculated for the in-session changes. More specifically, in-session changes were calculated for each session (changes from before to after session 1, etc.) as well as from before session 1 to after session 3.

## Results

### Participants

Eight patients with OCD were included in the present case series, four of them identified as female and four as male. Age ranged from 19 to 68 years with a mean age of 35. Comorbidities included adjustment disorder (12.5%), social phobia (12.5%), schizoaffective disorder (12.5%), substance use disorder (12.5%), impulse control disorder (12.5%), and major depression (37.5%).

### Feasibility

Retention for VRT-OCD was 100%; there were no drop-outs. Regarding intervention delivery and practicability, both therapists provided nearly exclusively positive feedback in the SUS. They stated that VRT-OCD could be learned quickly, that they felt confident using it, and that they could easily imagine using it regularly. However, one therapist indicated a need for technical support when using VRT-OCD, and another did not find the avatar therapy easy to implement without practice.

For all patients (100%), the therapists found the avatar beneficial and effective in conveying the session content (ATEST). Only in one case (12.5%), the therapist indicated that the therapy goals were not fully reached. They reported no negative side effects on the patient-therapist relationship and would recommend the use of VRT-OCD to other therapists. Overall, the therapist with more experience in VRT-OCD felt more secure with administering the treatment and was less critical about the intervention.

### Safety

**Figure 2.**
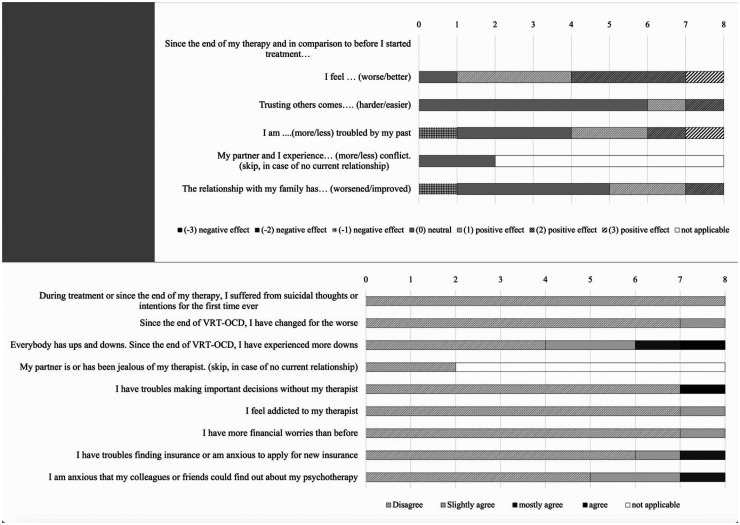
INEP side effects.

*Note*: INEP side-effects scales (Part 1 and Part 2). Displayed are the number of positive/negative/neutral responses from the eight participants (1–8 on *x*-axis). Green colour indicates positive feedback for the intervention.

The results on side effects (INEP) were largely neutral to positive (see Figure 2), indicating few negative side effects during or after the termination of VRT-OCD. There was no reported suicidal ideation during or after VRT-OCD. Seven out of eight participants felt better after the therapy than before (87.5%), while one participant did not notice any difference (12.5%). There were some reports of phases of worsening well-being (50.0%), increased fear that others might discover they were in therapy (37.5%), or concerns about health insurance issues (25.0%). However, participants assigned these negative experiences to other factors, not to VRT-OCD. Finally, there were no reports of any disrespectful, unprofessional or invasive therapist behaviour (assessed with the Therapeutic Misconduct subscale of the INEP).

### Acceptability

Based on the SARS, all participants (100%) agreed that VRT-OCD was useful, enjoyable, and would provide long-term benefits. They also stated they would recommend VRT-OCD to others (100%). None of the participants felt that the therapy negatively impacted other therapeutic interventions, caused disorganized thinking, or worsened their mood. Despite the generally positive feedback, some participants indicated challenges, including a reluctance to participate (25%). One participant felt that the therapy did not positively impact other treatments and did not consider VRT-OCD to be an important part of their overall care (12.5%). A visual display of the SARS results can be found in the supplements (B).

The SARS also included open questions asking for feedback regarding VRT-OCD. Patients liked it to literally face their OCD and to stand up to it. Multiple participants emphasized the importance of preparing the dialogue and what to say exactly before entering the VR. Regarding suggestions for improvement, participants wished for more sessions, the option to create a non-human avatar and an improvement of the sound quality of the voice modulator. A table with positive participant feedback can be found in the supplements (C).

### Preliminary treatment effects

**Figure 3.**
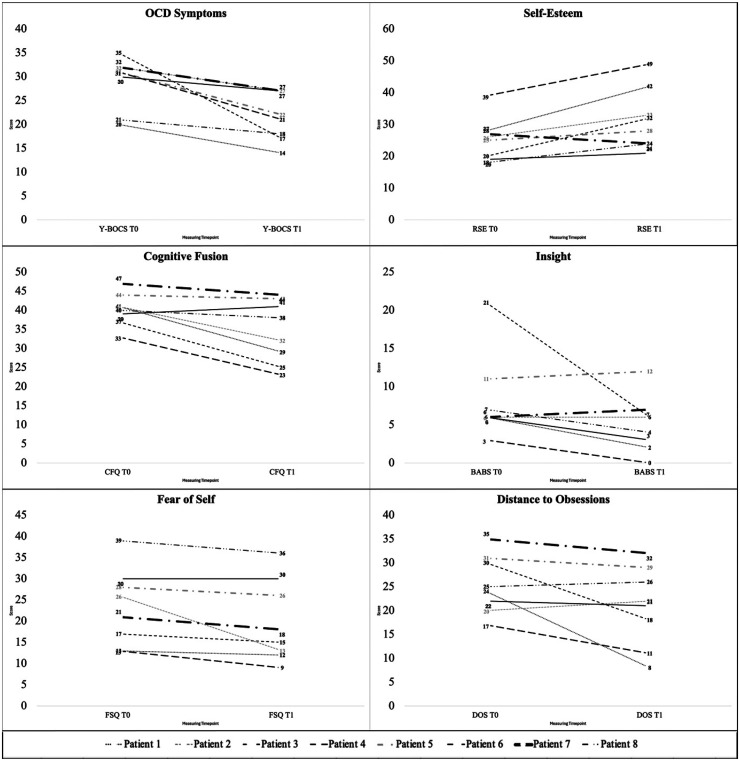
Changes in symptoms and associated constructs from baseline to post.

*Note*: BABS: Brown Assessment of Beliefs Scale; CFQ: Cognitive Fusion Questionnaire; DOS: Distance to Obsessions Scale; FSQ: Fear of Self Questionnaire; RSE: Rosenberg Self-Esteem Scale; Y-BOCS: Yale-Brown Obsessive Compulsive Scale. T0 = baseline, T1 = one week after the last VRT-OCD session.

Overall, we observed positive changes in OC symptoms and associated constructs (Figure 3). The sample showed a reduction in the Y-BOCS score with a large effect size (*d* = 0.90), while BABS scores decreased with a medium effect size (*d* = 0.66), indicating an increase in insight. A large effect size was found for the positive changes in the RSE (*d* = 0.74). The FSQ (*d* = 0.85), CFQ (*d* = 0.75) and DOS (*d* = 0.64) showed improvements with a medium effect size. Observing the single cases, the level of change seems rather homogeneous, in that all participants did show an improvement over the various measures. However, it appears that patients 4, 5, 7, and 8 only indicated minor changes, while patients 1, 2, 3, and 6 displayed stronger changes across all measures. While we saw a floor effect in the BABS scores (most participants had good insight at baseline), patient 3 evidently showed the strongest improvement in insight.

### In-session data

**Figure 4.**
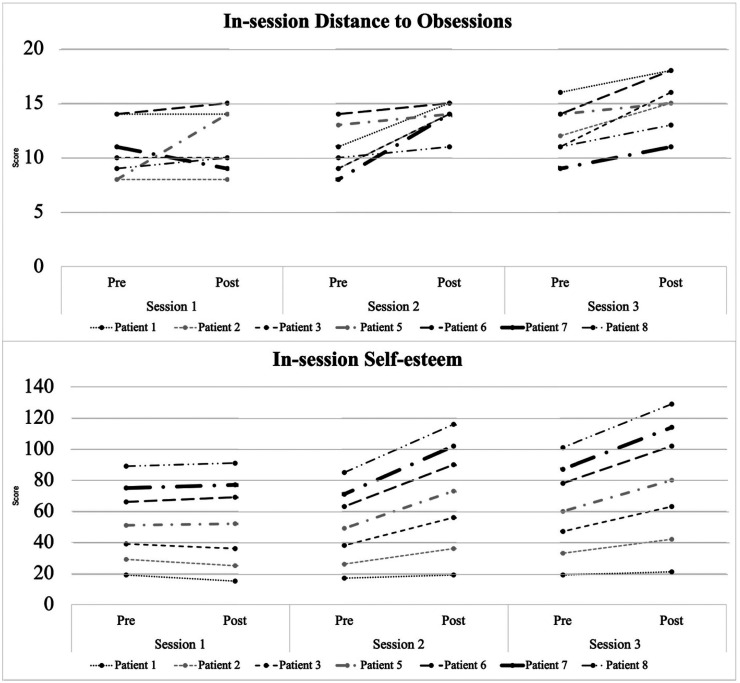
Changes in self-esteem and distance to obsessions in the session.

*Note*: In-session scores from patient 4 were excluded due to missing data.

The in-session data is displayed in Figure 4. Overall, scores on the in-session distance to the obsessions and self-esteem questionnaires increased over the course of the three sessions. Interestingly, we observed a slight decline between sessions (especially for self-esteem changes).

Looking at changes in distance to obsession, there was no significant effect in changes in the first session (*d* = 0.15). However, there was a medium effect in the second session (*d* = 0.78) and a large effect in the third session (*d* = 0.84). The effect size for changes in distance to obsessions from before session 1 to after session 3 was large (*d* = 0.84).

Regarding self-esteem, there was only a small effect in the first session (*d* = 0.26), but a large effect in the second (*d* = 0.85) and third sessions (*d* = 0.9). The effect size for changes in self-esteem from before session 1 to after session 3 was medium (*d* = 0.79).

## Discussion

The aim of this study was to assess the feasibility, safety, acceptability and preliminary treatment effects of VRT-OCD. By using a visual avatar to represent the disorder, the approach seeks to empower patients to resist their symptoms and enhance their insight into the nature of the condition. We assessed VRT-OCD in a case study including eight patients with OCD. While the initial results suggest safety, feasibility, acceptability, and positive treatment effects, the results must be regarded as preliminary and should be seen as the first clinical experience to generate hypotheses for future research.

### Feasibility, safety and acceptability

Overall, the feedback of participants and therapists indicates VRT-OCD to be a feasible, safe, and well-accepted intervention. There were no drop-outs, no negative side-effects evoked by VRT-OCD, no occurrence of suicidal ideation or worsening of symptoms (incl. mood), which are deemed highly relevant aspects in assessing the safety of novel treatments.^31^ Therapists found VRT-OCD easy to learn and were confident in its application after the training they received. Furthermore, despite the at times harsh words the therapists spoke to the patients when impersonating the avatar, there was no negative impact on therapeutic alliance. This is a crucial point, as rupture between patient and therapist can have such a detrimental impact on treatment outcome.^32^ Therapists highlighted the need for supervised training before applying VRT-OCD. Notably, the therapist with the most experience in VRT-OCD assessed the intervention more positively and expressed more confidence in its application. It should also be noted that the respective software is commercially available but not yet covered by insurance or public healthcare reimbursement programs.

Further, although all participants found the therapy beneficial, 25% of the participants had to force themselves to attend therapy, which may be due to the confronting nature of the intervention. Still, the participants did agree that VRT-OCD was rewarding. Further, in established approaches such as ERP, the confrontation and triggering of distress during therapy has a positive effect on symptom improvement.^4^ Thus, the challenging nature of VRT-OCD may not pose a problem, as long as participants are able to overcome the resistance they experience. This process may even be seen as a part of the therapy process. Nonetheless, future research on this treatment should assess reluctance to participate in ERP and drop-out rates. Additionally, it may be advisable to allow flexibility in the pacing of confrontation intensity, always in agreement with the patient's needs.

### Preliminary treatment effects

Participants demonstrated reductions in OCD symptoms and improvements in self-esteem, insight, fear of self, cognitive fusion, and their ability to differentiate themselves from their OCD. Although these changes could be influenced by regular inpatient treatment, the in-session data indicates immediate effects of VRT-OCD, with immediate improvements in both distance to obsessions and self-esteem. The observed decrease in distance to obsessions and self-esteem in-between sessions highlights the importance of repetition, aligning with recent findings on OCD treatment mechanisms by Miegel et al.^33^ Further exploration is needed to understand these fluctuations between sessions.

Distance to obsessions improved even more in session 3 than session 2, indicating a cumulative benefit from repeatedly facing the OCD and standing up to it. Self-esteem increased similarly across both sessions, despite only the third session explicitly targeting it, implying that standing up to OCD may inherently support self-esteem. These trends align with participants’ feedback requesting more sessions and warrant further investigation into the intervention's mechanisms of change.

### Externalization and insight

Externalizing OCD – visualizing it as separate from the self – may enhance insight into the disorder. This approach aligns with therapeutic practices like chairwork in Gestalt therapy^13^ or Internal Family Systems^14^ which focus on addressing different parts of the self. It also aligns with the concept of cognitive defusion in ACT,^18^ which teaches patients to detach from their thoughts and observe them rather than to take them as truths. The improvements in distance to obsessions, especially in-session, as well as defusion and insight seem to support this. While most participants had good baseline insight, one participant showed large improvements on the BABS, suggesting VRT-OCD may be especially helpful for individuals with poor insight. A separate case study explores this specific participant and the possible mechanisms in VRT-OCD related to insight.^20^

### Self-esteem

Self-esteem improvements were among the most significant changes reported by participants. While other interventions may have contributed, in-session data suggest a unique role of VRT-OCD. Participants emphasized the empowering effect of standing up to their OCD and reclaiming control. Preparing the dialogue – with therapist support – was noted as crucial, helping participants feel safe and confident during these confrontations.

Experiencing that one can respond differently to the OCD may constitute an experience of agency and mastery, which may in turn positively affect self-esteem. In this context, self-esteem seems to reflect a confidence in being able to navigate OCD-related uncertainty. Notably, although only the second VR dialogue targeted self-esteem, in-session data already showed increases in self-esteem after the first dialogue. The experience of successfully standing up to the avatar may be associated with improvements in self-esteem.

### Strengths and limitations

To the best of our knowledge, this is the first case series assessing VRT-OCD, offering preliminary support for its safety and acceptability. Further, a wide array of ages as well as comorbidities was represented, indicating that VRT-OCD may be suitable for a broad range of patients. The outcomes also imply the adaptability of VRT, suggesting the approach could be modified for various disorders such as PTSD or anxiety. Finally, integration of VR potentially adds accessibility for individuals with limited imagination- or visualization skills.

Nonetheless, we want to point out several limitations. The small sample limits generalizability, and the lack of a control group precludes from conclusions about efficacy. A small sample was chosen intentionally to assess feasibility, safety and acceptability before allocating larger funds. Further, all patients received concurrent inpatient treatment – while this ensured participant safety, it is unclear to what extent changes can be attributed to VRT-OCD. Additionally, our data does not allow any conclusions regarding which components of VRT-OCD specifically are associated with positive change in symptomology. Moreover, we did not assess any long-term effects – something that should be addressed in future studies on VRT-OCD. Lastly, while insight improved, most participants had good baseline insight, leaving open the question of the effects of VRT-OCD in this regard. Future research should employ larger samples, randomized controlled design and further explore the effects of the intervention, especially regarding insight.

### Clinical implications

If validated in the future, VRT-OCD may serve as a complementary treatment for patients with OCD, especially for those with low insight. The process of externalizing the OCD and actively challenging it may enhance motivation and treatment engagement, for example, for ERP. Future iterations could include modifications to optimize treatment outcomes (e.g. insight, OC symptom improvement), such as additional sessions, and a non-human avatar. Non-human representations may facilitate externalization by enabling patients to represent their OCD in a way that feels personally meaningful, potentially enhancing psychological distance from obsessions.

## Conclusion

In conclusion, VRT-OCD is a novel and promising approach to treat individuals with OCD. The intervention is feasible, safe, well-received by patients and therapists, and shows potential to improve symptoms and associated constructs such as insight and self-esteem. While the initial findings of this study are promising, the results should be interpreted with caution due to some discussed methodological limitations. Although further research is necessary, especially with larger samples and longer follow-up periods, the initial findings suggest that VRT-OCD has the potential to offer a valuable adjunct to existing OCD treatments.

## Supplemental Material

sj-docx-1-dhj-10.1177_20552076261434822 - Supplemental material for Meeting OCD face-to-face: Preliminary findings from an avatar-based dialogue intervention Supplemental material, sj-docx-1-dhj-10.1177_20552076261434822 for Meeting OCD face-to-face: Preliminary findings from an avatar-based dialogue intervention by Lara Wille, Luzie Lohse, Steffen Moritz, Josephine Schultz, Swantje Borsutzky, Amir H. Yassari, Lise Mariegaard, Ditte Lammers Vernal, Sanne Helene Bekker, Louise Birkedal Glenthøj, Franziska Miegel and Lena Jelinek in DIGITAL HEALTH
