# Supplementary material for: Meeting OCD face-to-face: Preliminary findings from an avatar-based dialogue intervention
Source: Digit Health. 2026 Apr 2;12:20552076261434822. doi: 10.1177/20552076261434822 (PMC13051170; doi:10.1177/20552076261434822)
Supplement: sj-docx-1-dhj-10.1177_20552076261434822 - Supplemental material for Meeting OCD face-to-face: Preliminary findings from an avatar-based dialogue intervention [file sj-docx-1-dhj-10.1177_20552076261434822.docx]

**Supplementary**

**Supplements A**

**Distance to Obsession Scale (DOS)**

To assess the extent to which the participant can externalize their obsessions, we developed the DOS. The scale consists of 6 items which are rated on a 7-point scale from 1 (*never applies)* to 7 (*always applies*). The questions in this scale majorly aim to capture whether the patient believes a) that their obsessive thoughts are part of who they are as a person, b) that their obsessive thoughts are true, and c) that the obsessions and compulsions help them to be safe. An example item is “*My obsessions are part of me*”. Possible sum scores ranged from 7 to 42, with lower scores indicating more distance from the obsessions. In this sample, the scale has Cronbach’s alpha of .7, indicating acceptable reliability.

**In-Session Instruments**

**Distance to Obsession In-Session (DOS In-Session)**

To measure changes in distance to the obsessions per session, we developed four change sensitive items that were filled out by the patients before and after each session. The items were answered using a 6-point Likert-scale ranging from 1 (*completely disagree*) to 6 (*completely agree*). The two reversed items we recoded so that a higher total score (ranging from 4 to 24) indicates more distance between the patient's healthy parts and their obsessive thoughts. An example item is “*I am allowed to do what’s important to me instead of listening to my obsessions*”. The reliability of this scale in this sample was acceptable with a = .7.

**Self-esteem In-Session**

To measure changes in self-esteem per session, we developed three change sensitive items that were filled out by the patients before and after each session. The items were answered using a 6-point Likert-scale ranging from 1 (*completely disagree*) to 6 (*completely agree*). The total score can range from 3 to 18, with higher scores indicating higher self-esteem. An example item is “*In this moment I feel confident*”. The reliability of this scale in this sample was acceptable with .71.

**Avatar Therapy Evaluation Questionnaire**

We developed these additional questions to assess to what extent patients found the implementation of a VR-Avatar as useful to their therapy. The four items are answered on a scale from 1 *(fully agree) to 5 (fully disagree).*

**Avatar Therapy Evaluation Scale for Therapists (ATEST)**

This nine-item scale was developed to ask about the suitability and added value of avatar therapy for the specific patient, as well as the personal evaluation of the therapy from the therapist’s perspective. Two of the items are open questions asking about likes and suggestions for improvements. In this sample, the scale displayed good reliability with an a of .82.

**Supplements B**

**SARS (Subjective Appraisal rating Scale).**

**
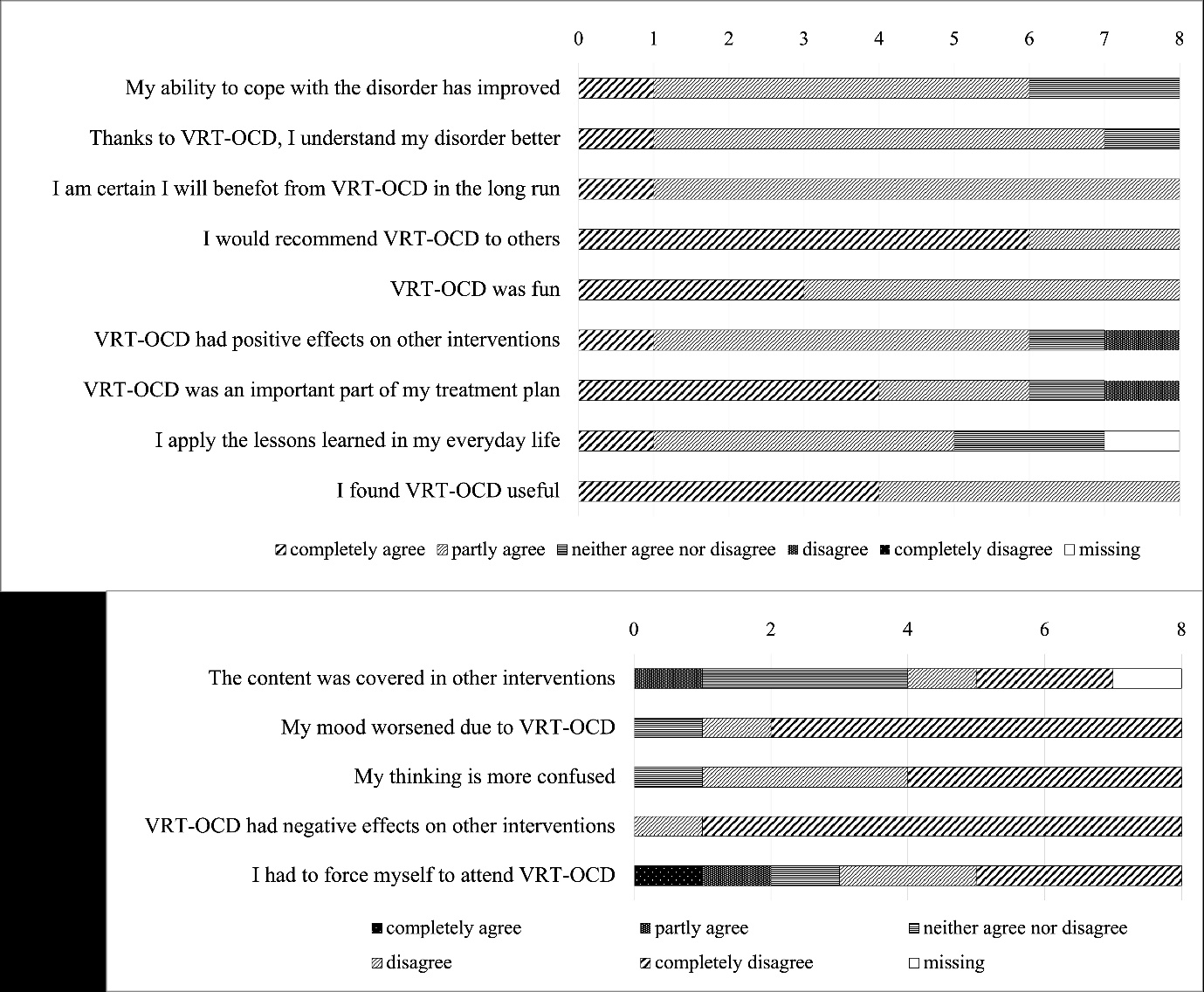
**

*Note*. Subjective appraisal of VRT-OCD at post-assessment (*n* = 8). The scale ranges from 0 to 100%. Positive items are displayed on top, negative items are displayed in the bottom half of the figure.

| **Supplements C**  **Participant Feedback** | |
| --- | --- |
| Patient nr. | Statement |
| 1 | I think it was good that we discussed how we would react to the OCD/avatar beforehand. I felt understood. |
| 2 | It's great that the OCD now has a face! It was good to fight back. It gave me a real reprieve. This energy also helped me with exposures! |
| 3 | Working out potential answers before the dialogue with the OCD enabled me to answer better and more courageously during the dialogue. I also liked the positive confirmation of my own answers during the conversation. |
| 4 | I really liked the innovative concept. The concrete formulation of the dialogue content and the associated engagement with it. |
| 5 | The dialogue with the externalized OCD.  The role play. Conversing with the OCD and standing up to it. |
| 6 | To tell the OCD that I can decide for myself + I don't need her/him, - To discuss my statements together (against the OCD). To say goodbye to the OCD! And to stand up against it with my own character traits. |
| 7 | Facing the OCD and being able to say strong words to him out loud/ being aware of these words.  To give the OCD a face/ to distinguish it from me. To identify his statements. |
| 8 | The conversation with the avatar about my positive qualities. |
| *Note*. Selected patient feedback from open questions SARS. Patient statements were translated from German to English. | |
